# Comparison of four commercially available ELISA kits for diagnosis of *Fasciola hepatica* in Irish cattle

**DOI:** 10.1186/s12917-019-2160-x

**Published:** 2019-11-21

**Authors:** Maria Pia Munita, Rosemary Rea, Ana Maria Martinez-Ibeas, Noel Byrne, Aideen Kennedy, Mary Sekiya, Grace Mulcahy, Riona Sayers

**Affiliations:** 1 Animal and Grassland Research and Innovation Centre (AGRIC), Teagasc, Moorepark, Fermoy, County Cork Ireland; 2 Biological Sciences, Cork Institute of Technology, Bishopstown, Cork, Ireland; 3 UCD School of Veterinary Medicine, University College Dublin, Belfield, Dublin 4 Ireland

**Keywords:** *Fasciola hepatica*, ELISA, Comparison, Sensitivity, Specificity, Treatment, Flukicides, Seasonality

## Abstract

**Background:**

*Fasciola hepatica* is a liver parasite of mammals and it results in poor welfare outcomes and economic losses in ruminants. While faecal egg count is the test most commonly used for diagnosis, it does not indicate presence of migrating immature stages. Serological techniques increase sensitivity at all stages of the liver fluke infection. The aim of this study was to compare four commercially available ELISA tests for the diagnosis of *F. hepatica*. For this purpose, we tested three sample types; (i) known *F. hepatica* status sera from an experimental infection for the comparison of sensitivities and specificities, (ii) sera from pre- and post-flukicide-treated (albendazole, closantel, nitroxynil and triclabendazole) beef cattle to contrast the differences of seropositivity before and after treatment, and (iii) bulk tank milk samples from dairy herds sampled during high and low *F. hepatica* exposure periods for assessing seasonal variations with the four tests available. Samples were tested using ELISA kits supplied by four manufacturers (Ildana Biotech, IDEXX, Svanova, and Bio-X). Samples were analysed simultaneously and in duplicate.

**Results:**

In the control population Ildana, IDEXX and Bio-X presented 100% sensitivity (Se) and specificity (Sp), Svanovir presented a Se of 59% and a Sp of 96%. In flukicide-treated beef cattle, kits highlighted decreasing antibody levels 90 days post-treatment in variable degrees. Finally, bulk milk showed a significant decrease in ELISA value between high and low fluke exposure periods with all tests studied.

**Conclusions:**

Se and Sp found in the present study, confirm that Ildana, IDEXX and Bio-X are accurate for the detection of *F. hepatica* exposure in Irish cattle. Svanovir Se and Sp in this population, indicate that a larger study is necessary to confirm this test characteristic in Irish herds. In post-treatment use, Bio-X showed a consistent and significant decrease of ELISA value in all groups treated, denoting to be a reliable tool for assessing treatment effect at 90 days post-treatment. Finally, all tests showed to be a reliable tool for the *F. hepatica* monitoring of high and low exposure seasons, using bulk tank milk samples.

## Background

*Fasciola hepatica*, commonly known as the common liver fluke, is a trematode parasite [1, 2] of mammals [3]. Its clinical manifestation is fasciolosis and it has a worldwide distribution [4, 5] reflecting a marked capacity for adaptation of both the causal agent and its intermediate mollusc host [5]. This adaptability, combined with the effects of global warming, increases the potential for *F. hepatica* related losses in livestock [6, 7] and increased prevalence in humans [5].

Fasciolosis is an important disease of domestic livestock [8] and both immature and mature stages of the parasite in the final host result in a 15% decrease in milk yield [9], an average reduction of 1.5 kg [10] or 0·7 kg milk/cow per day [11]. Annual losses have been estimated to be around €2.5 billion to livestock and food industry worldwide [12]. The presence of *F. hepatica* may also impact the shedding of *Escherichia coli* O157 in cattle destined for the human food chain [13]. Although livestock fasciolosis does not correlate with human fasciolosis [14], veterinary public health measures and food safety practices are recommended to reduce the risk of infection [5].

*Fasciola hepatica* has a preference for temperate climatic zones as its 18 to 30 week life cycle [4] requires mild temperatures and high humidity for the development of the intermediate host and free-living stages [15–18]. It can, however, also be found in areas of the tropics in conjunction with *Fasciola gigantica* [14]. The requirement for specific weather patterns for completion of its lifecycle leads to seasonal variations in livestock infection [19]. In temperate climatic zones without large seasonal climatic variations such as Ireland, management factors strongly influence the exposure and spatial distribution of the parasite [20, 21].

The definitive diagnostic test for *F. hepatica* is liver necropsy, which provides a highly accurate diagnosis of fasciolosis when bile ducts are dissected [22]. This is not practical as a herd or flock management tool as it can only be carried out post-mortem [23]. The most frequently used ante-mortem diagnostic test is the detection of eggs in faeces by sedimentation or flotation techniques, which are expressed as faecal egg count (FEC) [4] and had shown to have high specificity, detecting current infection [24], however, the accuracy of detection of small numbers of eggs in faecal samples is determined by the volume of sample available [22, 25] which constitutes a difficulty for diagnosis. The test can also be a poor indicator of infection when the parasite burden is low or when non-reproducing immature stages are migrating [26, 27], although increasing sample size and repeated sampling can increase both specificity (Sp) and sensitivity (Se). These diagnostic tools are laborious, time consuming, require skills for the identification of eggs and immature flukes, and are also unsuitable for a large scale or herd level testing [27, 28].

To prove effective as part of a control programme, diagnostic methods for herd screening must be reliable, easy to perform [28], and the cost of testing must relate to the benefit obtained by the diagnosis. Ideally, the diagnostic test should allow for early diagnosis of infection, and have the capability to detect seasonal differences in infection thus informing treatment decisions [29]. To meet these requirements, liver fluke specific enzyme-linked immunosorbent assays (ELISA) have been developed and are being routinely used in cattle [19, 30, 31]. *F. hepatica* ELISAs are adaptable tests which detect specific antibodies or antigens in faeces and pooled or individual milk and sera. Failure to diagnose immature migrating stages of liver fluke in the final host is a disadvantage of faecal egg counts, therefore the use of ELISA tests with the capacity of early diagnosis is a major advantage. The most damaging stage of this infection in the final host occurs during the migration of immature stages. The use of ELISA techniques for *F. hepatica* diagnosis has demonstrated improved sensitivity of diagnosis over coprological techniques, and has the improved advantage of detection of pre-patent infections [11, 31, 32]. Additionally, detection of *F. hepatica* antigens in faeces is also feasible; using available copro-antigen ELISA kits, which have shown to have high sensitivity and specificity [31, 33].

At present, several *F. hepatica* ELISA kits for sera and milk are commercially available, each comprising of different antigens, methods, sample dilutions, S/P% calculations and thresholds. Comparison of these tests under identical conditions has not been previously assessed and the wide range of commercial ELISA tests generates indecision in the related community as to which test to use and the significance of results. It is important to *F. hepatica* management in cattle herds that commercially available kits can not only detect infection but can do so in a timely manner with the ability to detect seasonal variations and post-treatment effects. The present study, therefore, aimed to evaluate and compare four commercially available ELISA kits for milk and sera, in their ability to detect exposure to *F. hepatica* in Irish cattle of known status and in naturally infected herds; pre-and post-flukicide treatment (sera), and in bulk tank milk (BTM) samples taken over a 12 months period.

## Results

### Assay sensitivity and specificity

In all, 24 pre-colostral samples and 44 experimentally infected samples (22 at four wpi and 22 at 10 wpi) were tested across both groups. No liver fluke eggs were evident in experimentally infected calves at 4 weeks post- infection. However, all infected calves recorded positive FECs by 10 wpi and infected livers post-mortem (data not shown).

**Fig. 1:**
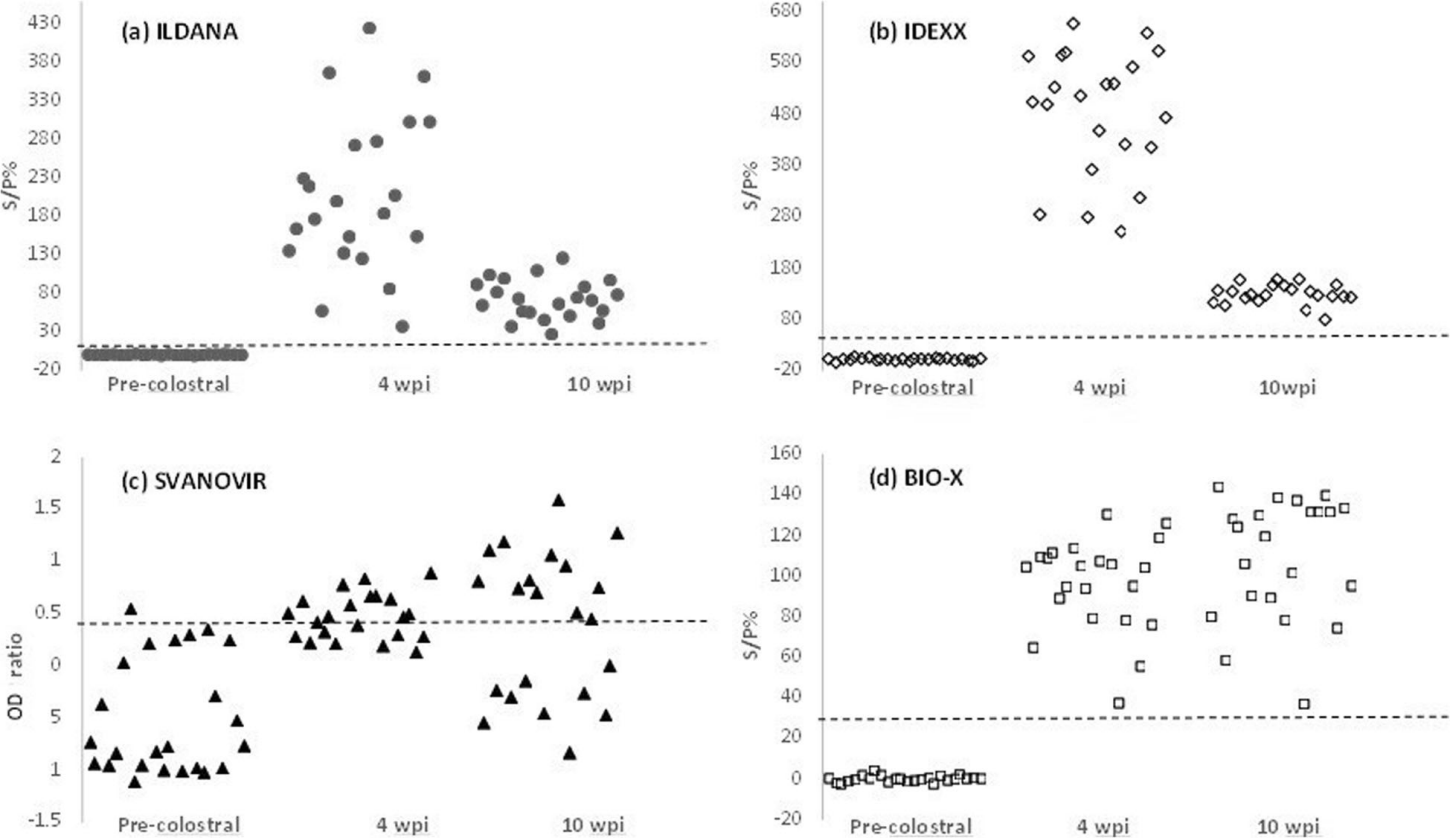
Scatter plots outlining ELISA results from pre-colostral and experimentally infected calves (4 and 10 weeks post-infection (wpi)) across (**a**) ILDANA, (**b**) IDEXX, (**c**) SVANOVIR and (**d**) BIO-X *F. hepatica* test kits. Positive cut-off values for each kit are represented by the dashed line (−---)

Results from pre-colostral and experimentally infected calves (4 and 10 wpi) across the four tests can be seen in Table 2 and Fig. 1. Of the kits examined Ildana, IDEXX and Bio-X correctly identified all 24 pre-colostral samples as negative and all 22 experimentally infected samples at both four and ten wpi as positive (Table 2). This yielded Se and Sp for Ildana, IDEXX, and Bio-X kits of 100 and 100%, respectively, from at least four wpi (Table 2). The Svanovir kit classified 23 of the 24 pre-colostral samples as negative and 13 of 22 experimentally infected samples at 4 and 10 wpi as infected with *F. hepatica* with likely production losses (Table 2). This yielded a Se of 59.1% and Sp of 95.8% for the Svanovir test (Table 2).

For all commercial kits examined, the number of positive and negative cases at four and ten wpi remained consistent. However, variations in S/P% and ODRs were observed principally in Ildana (*P* ≤ 0.0001), IDEXX (*P* ≤ 0.0001) and Svanovir (*P* ≥ 0.05) (Fig. 1), although all samples remained clearly positive. These variations in Ildana and IDEXX were characterised by a decrease in the range of positive S/P% (i.e. Ildana- 4wpi: 30 to 400%, 10wpi: 30 to 130%; IDEXX- 4wpi: 200 to 590%, 10wpi: 80 to 170%) (Fig. 1). In contrast, the Svanovir test showed an increase in this range (4wpi: 0.4ODR to 0.8ODR, 10wpi: 0.4ODR to 1.5ODR), but this change was not significant (Fig. 1). No evident or significant changes were observed with the Bio-X kit.

### Pre- and post-treatment kit variations in naturally infected beef cattle

**Fig. 2:**
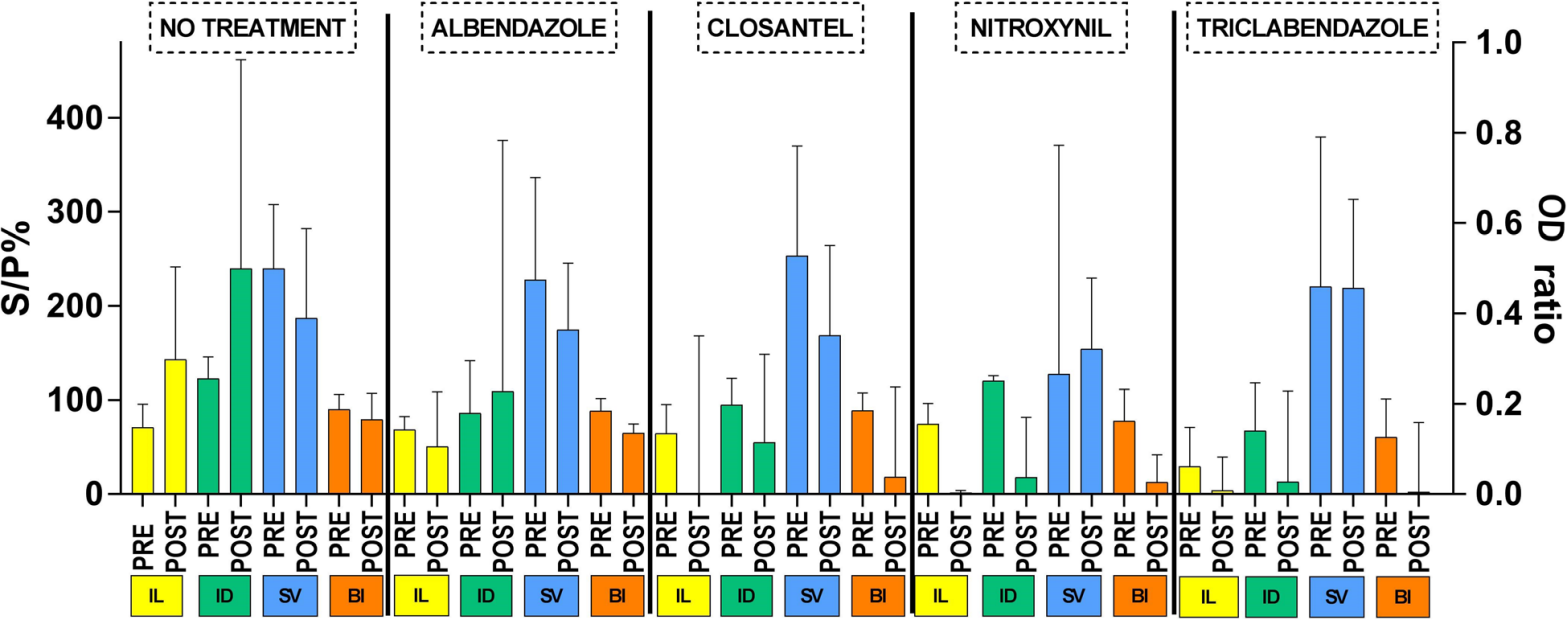
Boxplot of pre- and post-treatment results from naturally infected beef cattle across test kit and five flukicide treatments. Ildana, IDEXX and Bio-X kits recorded results as S/P% (left Y axis) and the Svanovir kit recorded results as ODR (right Y axis). IL = Ildana Biotech *Fasciola* ELISA test kit. ID = IDEXX *Fasciola hepatica* antibody test kit. SV = SVANOVIR *Fasciola hepatica* antibody test. BI = Bio-X Diagnostics *Fasciola hepatica* ELISA kit. Pre = value prior to treatment i.e. Day 0. Post = value 90 days post-treatment i.e. Day 90

Ten animals were allocated to each treatment group and in total 50 individual sera samples were tested on two occasions (before and after the administration of a flukicide treatment). A boxplot outlining pre- and post-treatment results for each test kit across the five groups investigated are included in Fig. 2.

Wilcoxon matched-pairs signed rank test for determining the significance of post- treatment variations determined significance in nine of the 20 comparisons (Table 3). The Bio-X test showed a decrease of S/P% in albendazole (*z* = 2.85, *P* < 0.01), closantel (*z* = 2.67, *P* = 0.01), nitroxynil (*z* = 2.76, *P* = 0.01) and triclabendazole (*z* = 2.76, *P* = 0.01) groups and for these groups, the decrease was observed in nine of the ten animals evaluated (Table 3). In contrast, the Svanovir test only showed a significant decrease of ODRs 90 days after albendazole treatment (*z* = 2.40, *P* = 0.02) (Table 3).

Ildana and IDEXX tests detected an increase of S/P% 90 days after first sample in the no treatment group (*z* = − 1.96, *P* = 0.05 and *z* = − 2.40, *P* = 0.02, respectively), this increase resulted from six and nine animals, respectively. Additionally, the Ildana test detected a significant decrease of S/P% in eight of the ten animals treated with nitroxynil.

### High and low exposure season detection in naturally infected bulk tank milk samples

A total of 103 BTM samples from 29 herds were analysed using the four ELISA kits. In all, 14 herds supplied samples for all 4 time points. The mean number of samples received per month was 26 samples (range 20 to 29). Herd sizes ranged from 60 to 310 milking cows, the mean herd size being 157 cows. All study herds were specialist dairy enterprises with no additional livestock species such as fattening of cattle or sheep on the farm. All cows were grazing from February until November and were housed in December and January.

**Fig. 3:**
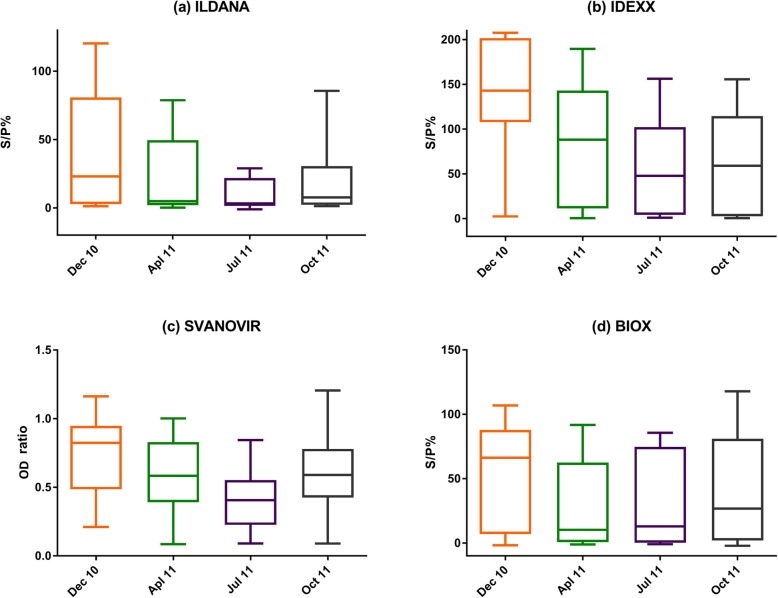
Box plots of bulk tank milk ELISA results across test kit (**a**) Ildana, (**b**) IDEXX, (**c**) SVANOVIR, (**d**) BIO-X and time (December 2010, April 2011, July 2011, October 2011)

Test results from the four ELISA kits revealed the highest S/P% and ODR medians in December 2010 and the lowest in July 2011 (Ildana (3.31 S/P%), IDEXX (47.81 S/P%) and Svanovir (0.41 ODR)), the Bio-X test showed the lowest S/P% in April 2011 (10.3 S/P%) (Fig. 3).

Generalized estimating equation (Table 4) confirmed the higher risk and lower exposure seasons observed in the previous descriptive analyses. All tests showed significant decreasing antibody levels in April, July and October against December. A decrease in July alongside April was significant with the Ildana, IDEXX and Svanovir test (*P* = 0.006, < 0.001 and 0.021, respectively). A significant decrease in S/P% was observed in October in comparison to April using the IDEXX test (Coefficient = − 27.04; *P* = 0.003), on the contrary the Svanovir test showed a small significant increase (Coefficient = 0.09; *P* = 0.044). Finally, higher S/P% were detected in farms which treated with a flukicide in contrast with farms which did not use any flukicide treatment.

## Discussion

The aim of this study was to compare and evaluate four commercially available ELISA kits (Ildana, IDEXX, Svanovir, Bio-X) for diagnosis of *F. hepatica* in Irish cattle, as a comparative study of the four tests available has not been previously reported. For this purpose, samples from three different populations were evaluated, including sera from a known-status population (known positive and negative sera), sera from naturally infected beef cattle (before and after treatment with different flukicides) and naturally infected BTM samples from 31 dairy herds (collected in four different months within a year). To evaluate these tests a paired design was applied, offering advantages like the minimisation of between-subject variability and elimination of confounding [29]. Also, the evaluation of these tests in naturally infected populations gives a real view in day-to-day parasite control measures practiced.

The Ildana assay is based on a recombinant antigen [34] (Table 1) and has previously been used in multiple *Fasciola hepatica* studies in Ireland [19, 21, 37]. Sensitivity and specificity ratios reported previously were 98% in bovine sera [37]. Previous studies, which also used recombinant antigens, have reported similar sensitivities and specificities at variable times post- infection [38–40], and no cross-reaction with other present parasites was observed [39]. The IDEXX kit (f2 antigen) (Table 1) (originally the Pourquier ELISA) has shown to be very reliable, as previous experimental infections reported sensitivity and specificity ratios of 100% [38, 41] and close to a 100% in natural infections [42, 43]. However, a study by Simões et al. in 2017 reported a specificity ratio of 56% in Brazil [44]. The E/S antigen, in the Svanovir kit (Table 1), has previously shown a strong correlation between *F. hepatica* antibody levels, intra-hepatic fluke frequency and production parameters [11, 45] and a sensitivity and specificity of 92 and 88%, respectively, in BTM samples compared to sera [30]. With regards to the Bio-X kit, containing the CL1 antigen (Table 1), an earlier study found a strong correlation between an in-house ELISA, which used the same antigen as Svanovir (E/S), and Bio-X in sera from non-infected and naturally infected cattle [46].

**Table 1:** Summary of ELISA kit characteristics

	ILDANA	IDEXX	SVANOVIR	BIO-X
Coating antigen	rmCL1	f2	E/S protein	MM3
Antigen source	Recombinant [17]	Purified from natural *F. hepatica* E/S products [34]	Purified from supernatant of *F. hepatica* incubation media [35]	Purified from supernatant of *F. hepatica* incubation media [36]
Sample types	Serum, milk, BTM	Serum, milk, BTM	Serum, milk, BTM, meat juice	Serum, milk, BTM
Serum sample dilution	1/20	1/20	1/100	1/100
Milk sample dilution	Not required	1/20	Not required	1/4
Total incubation	70 min	110 min	150 min	130 min
Optical density	450 nm	450 nm	405 nm	450 nm
Serum Positive Cut-off	≥ 15S/P%	> 30S/P%	≥ 0.4ODR^a^	≥ 10S/P%

^a^ODR above which there is infection with liver fluke and likely associated production losses. Values below this cut-off indicate no or low fluke burdens

**Table 2:** Classification of samples from pre-colostral and experimental infected animals at 4 and 10 weeks post-infection (wpi) by four commercial *F. hepatica* test kits

Assay	Classification	Exposed 4 wpi	Exposed 10 wpi	Not exposed	Kit sensitivity (%)	95% confidence interval	Kit specificity (%)	95% confidence interval
ILDANA	Positive	22	22	0	100.0	84.6, 100.0		
	Negative	0	0	24			100.0	85.8, 100.0
	Total	22	22	24				
IDEXX	Positive	22	22	0	100.0	84.6, 100.0		
	Negative	0	0	24			100.0	85.8, 100.0
	Total	22	22	24				
SVANOVIR	Infected	13	13	1	59.1	36.4, 79.3		
	Low or no infection	9	9	23			95.8	78.9, 99.9
	Total	22	22	24				
BIO-X	Positive	22	22	0	100.0	84.6, 100.0		
	Negative	0	0	24			100.0	85.8, 100.0
	Total	22	22	24

**Table 3:** Wilcoxon matched-pairs rank-signed analysis of individual cow pre- and post-treatment serum ELISA S/*P* values across four test kits and five flukicide treatments

Test	ILDANA		IDEXX		SVANOVIR		BIO-X	
Flukicide treatment	Median Day 0 vs. Day 90^a^		Median Day 0 vs. Day 90^a^		Median Day 0 vs. Day 90^a^		Median Day 0 vs. Day 90^a^	
	*z* value	*P* value	*z* value	*P* value	*z* value	*P* value	*z* value	*P* value
	(*n* = 10)		(*n* = 10)		(*n* = 10)		(*n* = 10)	
No treatment	71.93 vs. 170.96		123.46 vs.282.60		0.54 vs.0.45		92.39 vs.83.23	
	−1.96	0.05	−2.40	0.02	2.05	0.04	1.69	0.09
	6↑ 4↓^b^		9↑ 1↓		2↑ 8↓		4↑ 6↓	
Albendazole	70.72 vs.51.38		81.13 vs.115.80		0.48 vs. 0.40		90.39 vs.67.87	
	0.36	0.72	−1.33	0.18	2.40	0.02	2.85	< 0.01
	2↑ 8↓		5↑ 5↓		2↑ 8↓		1↑ 9↓	
Closantel	68.13 vs.1.09		104.96 vs. 66.07		0.57 vs. 0.37		100.30 vs. 30.11	
	0.09	0.93	1.51	0.13	1.78	0.08	2.67	0.01
	3↑ 7↓		4↑ 6↓		4↑ 6↓		1↑ 9↓	
Triclabendazole	56.59 vs. 0		76.27 vs.15.26		0.49 vs. 0.46		68.06 vs.2.88	
	1.69	0.09	1.38	0.17	0.89	0.37	2.76	0.01
	2↑ 8↓		2↑ 8↓		5↑ 5↓		0↑ 10↓	
Nitroxynil	78.32 vs.1.31		122.61 vs. 24.79		0.28 vs. 0.32		91.74 vs. 16.01	
	1.96	0.05	1.78	0.08	0.53	0.59	2.76	0.01
	2↑ 8↓		4↑ 6↓		6↑ 4↓		1↑ 9↓	

^a^Day 90 indicates 90 days post-treatment with a particular flukicide

^b^Indicates the number of animals per group that either increased (↑) or decreased (↓) in S/P result

**Table 4:** Generalized estimating equation analysis of bulk tank milk continuous ELISA results

Independent variable	Coefficient (*n* = 31)	Confidence interval (95%)	*P* value	Model *P* value
ILDANA				
April vs. December	−24.21	−34.09, −14.32	< 0.001	Time Herd size Treatment (< 0.001)
July vs. December	−36.46	−46.37, −26.55	< 0.001	
October vs. December	−21.97	−32.30, − 11.65	< 0.001	
July vs. April	−12.25	−20.91, −3.59	0.006	
October vs. April	2.23	−6.79, 11.25	0.628	
October vs. July	14.48	5.42, 23.54	0.002	
Treated vs. not treated	24.17	6.62, 41.72	0.007	
IDEXX				
April vs. December	−36.22	−55.98, −16.47	< 0.001	Time Herd size Treatment (< 0.001)
July vs. December	−70.78	−90.57, − 50.98	< 0.001	
October vs. December	−63.27	−83.91, −42.63	< 0.001	
July vs. April	−34.55	−51.82, −17.27	< 0.001	
October vs. April	−27.04	−45.07, −9.02	0.003	
October vs. July	7.50	−10.57, 25.58	0.416	
Treated vs. not treated	29.69	−10.30, 69.69	0.146	
SVANOVIR				
April vs. December	−0.19	−0.28, −0.10	< 0.001	Time Herd size Treatment (< 0.001)
July vs. December	−0.29	− 0.38, − 0.19	< 0.001	
October vs. December	− 0.10	− 0.20, − 0.01	0.036	
July vs. April	− 0.09	− 0.17, − 0.01	0.021	
October vs. April	0.09	0.01, 0.17	0.044	
October vs. July	0.18	0.09, 0.26	< 0.001	
Treated vs. not treated	0.11	−0.04, 0.26	0.162	
BIO-X				
April vs. December	−22.00	−31.54, −12.47	< 0.001	Time Herd size Treatment (< 0.001)
July vs. December	−18.75	−28.30, −9.20	< 0.001	
October vs. December	−16.41	−26.37, −6.45	0.001	
July vs. April	3.25	−5.07, 11.57	0.444	
October vs. April	5.59	−3.09, 14.28	0.207	
October vs. July	2.34	−6.36, 11.05	0.598	
Treated vs. not treated	30.07	20.29, 91.31	0.002	

In the known status population all tests detected *F. hepatica* antibodies 6 weeks prior to the detection of eggs by FEC. This early diagnosis has been widely described in the literature [38, 41] and the present study confirms this characteristic for the four different commercial tests assessed. Ildana, IDEXX and Bio-X tests presented a 100% Se and Sp in an experimental population and is in line with sensitivity and specificity ratios previously reported. Although the Svanovir test did not reach the 100% Se and Sp it showed to be suitable for the use with BTM samples for determination of in-herd prevalence and seasonal changes. In the present study, the Svanovir test detected one naïve animal as positive, suggesting a possible attachment of unspecific antibodies to the antigen. Cross-reaction could only be possible in the presence of other worm infections [38] and sera used as known status- negative to *F. hepatica* were collected from naïve animals, with no previous exposure to pasture helminths (pre- colostral). This finding suggests that further research needs to be conducted with this test in Irish cattle as no previous reports of this finding are available in the literature.

A decrease in S/P% was observed with the Ildana and IDEXX kits (Fig. 1) at 10 weeks post-infection suggesting final stages of the primary immune response [47], however, further research is needed to confirm this. The decrease in S/P% observed in the present study has previously been reported with IDEXX, with positive cases still remaining positive after treatment [41] as in the present study. But IDEXX has also shown to maintain constant antibody detection after shorter periods of time (21 to 42 days post- infection) depending on infection dose [38]. In the present study, Svanovir and Bio-X showed more stable S/P% and ratios at 10 weeks post- infection (Fig. 1), the differences observed in ODR at 10 weeks post infection could be explained by variations in the stability of the binding reaction between antigen and antibody [48], however, further study would be needed to confirm this observation.

As natural infections are usually constant during high risk grazing periods [19], adult animals present higher levels of detectable antibodies as they have been exposed to more high risk seasons. Having said that, the experimental infection method used in the present study included a single infective dose, containing 115 METs and was administered to young animals. Experimental infection does not necessarily equate the response measured by these tests in adult cow populations, as adult cows have been exposed repeatedly through their productive lives, with the possible build-up of specific antibodies. This is of special importance in pasture-based systems, like Ireland.

In general, the naturally infected-beef population presented decreases of ELISA values 90 days after the application of treatment. The Svanovir kit has previously shown a significant decrease of ODRs at 3–6 months [49] and 1 year [50] post treatment. These previous reports and the present results propose the use of the Svanovir kit more than 90 days after treatment. In comparison, results obtained from the Bio-X test confirmed that a 90 days period after treatment is adequate to measure its effects.

In the naturally infected populations studied, the four kits showed a general agreement in the detection of *F. hepatica* antibodies in the different groups, this effect was especially evident in the BTM population. The Svanovir test discrepancies seen in the known status population were not evident in the BTM sample group, this could be attributed to a larger sample size, the dilution of antibodies in bulk tank milk samples [51] or higher concentration of antibodies in adult animals.

Changes in BTMs antibody detection were dependent on the seasonal exposure variations (Fig. 3 and Table 4), as previously described in Europe [15], Germany [52] and Ireland [19], which defined winter as high exposure season and summer as low. Conventionally, ELISA testing has been used with individual sera or even with pooled sera for herd diagnosis. However, ELISAs are being widely used on BTM samples [19, 51] because of the practicality for the determination of herd-level status, making the BTM antibody ELISA an attractive alternative [30, 31]. The inconvenience of the use of ELISA tests for the detection of *F. hepatica* antibodies is that results do not necessarily indicate the presence of active infection, as antibodies will still circulate after treatment [51] and the levels of exposure would also be related with age and stage of the milking period, it is important to consider the treatment measures applied, age and milking period of the herd before interpreting BTM ELISA results. Nevertheless, the four tests studied detected the classical seasonal dependent variations on antibody levels in Irish dairy herds (Fig. 3).

## Conclusions

It is clear that fasciolosis represents a major risk for the health and production of cattle worldwide, moreover, a potential increase of *F. hepatica* burden has been predicted as a result of climate change [7]. For the appropriate treatment and control of liver fluke, diagnosis is key. As previously mentioned, the diagnostic test should allow the early diagnosis of the disease, be able to detect seasonal differences in infection and thus informing treatment decisions [29]. The present study highlights the early and reliable diagnostic capacity of the four commercially available tests assessed for fasciolosis, although, the Svanovir kit presented lower sensitivity and specificity under experimental conditions. For a better understanding of the Svanovir results in relation with sensitivity and specificity, further studies need to be performed using the Svanovir test in Irish cattle. All tests detected changes in antibody levels 90 days post-treatment and Bio-X showed greater accuracy in this detection as all changes after treatment were significant. However, a larger sample population and/or longer sampling time would be necessary to confirm the findings using the Svanovir kit as observed by Köstenberger et al. in 2017 [50] and Charlier et al. in 2012 [49]. The use of all four tests with BTM samples showed to be a reliable tool for the determination of high and low exposure seasons and in-herd prevalence throughout the year, however, results must be interpreted considering herd health management, *Fasciola hepatica* life cycle and herd milking pattern.

## Methods

### Sample populations

#### Calves of known *F. hepatica* status – control population

To source negative samples of known *F. hepatica* status, blood from 50 neonatal pre-colostral, housed calves born in January and February 2016, were collected into plain vacutainers. Calves were either Holstein-Friesian or Jersey-cross breeds. All blood samples were collected within the first hour post-calving. These calves were born and housed at Teagasc (Irish Agriculture and Food Development Authority), Moorepark, County Cork, Ireland. Samples were collected by a parallel study under license from the Health Products Regulatory Authority (HPRA) (AE19132/P044) and approved by the Teagasc Animal Ethics Committee (TAEC).

For the purposes of obtaining positive samples of known *F. hepatica* status, blood samples were collected from Holstein-Friesian calves (*n* = 25) experimentally infected with *F. hepatica* metacercariae (MET). Experimental infection was achieved by orally dosing each calf with 115 METs, after 10 weeks grazing period, this pre-infection grazing period was carried out for acclimatisation and ensuring infection. Calves were housed immediately post-infection. Grazing and housing took place at Teagasc farm. These animals were infected as part of a 75-calf vaccination trial funded by the Irish Department of Agriculture, Food and the Marine and licensed by the HPRA (AE18982/P088) and TAEC. Only non-vaccinated calves (control infected group) were included in the current study. Blood and faecal samples were collected on the fourth week post infection (wpi) for assessing the detection of immatures stages and on the tenth wpi for assessing mature parasites. For the purpose of post-mortem evaluation, infected animals were transported and slaughtered according to the Irish Slaughter of Animals Act [53] 3 months post-infection.

#### Naturally infected herds - blood

Blood samples were collected from a commercial beef herd, containing animals of varying beef breeds, crossbreeds and ages, located in county Clare, Ireland. This herd recorded a prior history of *F. hepatica* infection by ELISA diagnosis (data not shown). Animals were housed for the winter months and grazed for the rest of the year, the current experiment was carried out during the housing period. Samples were initially collected and analysed for the purposes of evaluation of dosing strategies in farmed beef in Ireland, and when archived, these samples were made available to the current study. Samples were available from five different treatment groups; albendazole (dose rate 10 mg per kg), closantel (dose rate 10 mg per kg), nitroxynil (dose rate 10 mg per kg), triclabendazole (dose rate 12 mg per kg) and a no treatment control group. Individuals were randomly assigned to each treatment group and each group contained 10 animals. Flukicides were administered based on body weight estimates, on a single occasion, and administered orally except for closantel which was a ‘pour on’ preparation. Blood samples were collected from the 50 individuals prior to dosing (Day 0; February 2016) and again 90 days post-treatment (Day 90; May 2016); to allow comparison of pre- and post-treatment ELISA results under farming conditions in Ireland. The dosing strategies experiment which supplied samples for the current study was approved by the TAEC and licenced by the HPRA (AE19132/P031) and funded by the Irish Department of Agriculture, Food and the Marine.

#### Naturally infected herds - BTM

Archived bulk tank milk (BTM) samples were available from 29 herds, 22 of which were commercial dairy herds and members of the Dairy Management Information System, a discussion group coordinated by Teagasc. The remaining 7 herds were Teagasc dairy research herds. Each herd was requested to submit four BTM samples; in December 2010, April 2011, July 2011 and October 2011. Herd sizes and *F hepatica* dosing frequency and active ingredient were available for each herd.

### Sampling methods

Blood samples from neonatal calves were obtained by jugular venepuncture. All other blood samples were collected using venepuncture of the coccygeal vein. BTM samples were collected by individual farmers and submitted to Teagasc by post in a standardised sampling kit [19]. On receipt at the laboratory, blood and BTM samples were centrifuged (4000 g for 4 mins, blood; 20,000 g for 1 min, BTM). Serum and skim BTM were subsequently collected into 1.5 mL microtubes and frozen at -80 °C until analysed, ensuring only one freeze/thaw cycle. Samples were obtained on the dates specified in the sampling populations sections of the present study. Faecal samples collected from the ground (faecal catch) from the experimentally infected group were stored in sample pots and analysed at the arrival to the laboratory.

### Sample analysis

Five grams of faeces were homogenised with water and passed first through a coarse mesh sieve and then a finer, 250 μm mesh sieve. The filtrate was allowed to stand for 5 min to sediment and the supernatant was removed by aspiration. Sedimentation was repeated 1–2 times as required. The supernatant was removed and the sediment was stained with two drops 1% methylene blue. Eggs were counted on a stereomicroscope as outlined by Taylor, et al., 2007 [4]. Results were expressed as presence or absence of liver fluke eggs and all samples were evaluated by the same person.

Blood and BTM samples were analysed concurrently using four commercially available ELISA kits; Ildana Biotech *Fasciola* ELISA test (Ildana Biotech, Ireland), IDEXX *Fasciola hepatica* antibody test kit (IDEXX, France), Svanovir *F. hepatica*-Ab (Svanova, Sweden) and Bio-X Diagnostics *Fasciola hepatica* Ab ELISA kit (Bio-X Diagnostics, Belgium). All testing was carried out by the same person. All kits have been validated by the manufacturers for use with individual milk, pooled milk and serum samples. Results for Ildana, IDEXX and Bio-X kits were expressed as sample to positive percentage (S/P%) and as optical density ratio (ODR) for the Svanovir test. The specific characteristics of each test are included in Table 1. All assays were completed following manufacturer’s instructions including calculation of S/P% and ODRs.

### Sample classification

In addition to continuous ELISA results, serological results were classified as positive or negative according to kit positive cut-off values for three kits (Ildana, IDEXX, Bio-X). The fourth kit (Svanovir) classified samples based on a cut-off value above which animals were deemed infected and whether the potential for production losses existed. These cut-off values are outlined in Table 1 for each assay examined.

Two further categorisations of dairy herds were completed for bulk tank milk analyses. Firstly, herds were classified as small (50 to 120 milking cows), medium (121 to 190 milking cows) or large (over 190 milking cows). These herd size ranges were defined to best represent the data recorded, generating categories of similar size. Secondly, herds were classified on whether or not a *F. hepatica* dosing protocol was applied in winter 2010.

### Statistical analyses

Microsoft Excel (MS Office, 2010) was used for data collation and initial descriptive analyses including scatter plots. Assay Se, Sp and associated statistics were calculated using the MEDCALC diagnostic test evaluation calculator (https://www.medcalc.org/calc/diagnostic_test.php). Normality of the data was assessed by Shapiro-Wilk normality test and visually using ladder of powers histograms constructed in Stata version 12 (StataCorp, USA). Boxplots, Wilcoxon matched-pairs signed-rank test and Generalised estimating equations (GEE) were completed using Stata version 12. GraphPad Prism 7 (GraphPad Software Inc., 2017) was used to construct the box plot of natural infection-blood data.

Three databases were created, one for each sampling population (known status, natural infection – blood and natural infection – BTM). The Se and Sp of each kit was calculated for each test using positive and negative samples of known status. Assay Se was calculated as the probability that an experimentally infected calf would be identified as positive (Ildana, IDEXX, Bio-X) or infected (Svanovir) with *F. hepatica*, based on manufacturer’s interpretation criteria. Sp was calculated as the probability that a pre-colostral calf would be identified as negative (Ildana, IDEXX, Bio-X) or not likely to have been exposed to *F. hepatica* (Svanovir).

The naturally infected – blood data from beef cattle were analysed by Wilcoxon matched-pairs signed rank test to examine whether significant differences existed between pre- and post-treatment groups across each assay. Additionally, a boxplot was generated to allow visualisation of results across both treatment and ELISA kit used.

To examine whether trends in seasonality could be detected using the assays under investigation, BTM data (continuous) were analysed by GEE. For all continuous GEE analyses, herd was included as a repeated measure and an exchangeable correlation used. A Gaussian family and identity link function was used. Independent variables included in the analyses were herd size (small vs. medium vs. large), dosing protocol (dosed vs. not dosed in winter 2010), and time (December 2010 vs. April 2011 vs. July 2011 vs. October 2011). These variables were forced into the model regardless of their significance level due to their potential impact on BTM results. Finally, results from herds that provided a complete set of four BTM samples were plotted against assay intermediate positive cut-off values to visualise seasonality across assays.

## Data Availability

All data is stored in the Teagasc (national food and development authority) database. The datasets used and/or analysed during the current study are available from the corresponding author on reasonable request.

## Abbreviations

BTM: Bulk tank milk
ELISA: Enzyme-linked Immunosorbent assay
FEC: Faecal egg count
GEE: Generalised estimating equations
HPRA: Health Products Regulatory Authority
MET: Metacercariae
ODR: Optical density ratio
S/P%: Sample to positive percentage
Se: Sensitivity
Sp: Specificity
TAEC: Teagasc Animal Ethics Committee
